# Influence of delivery and feeding mode in oral fungi colonization – a systematic review

**DOI:** 10.15698/mic2020.02.706

**Published:** 2020-01-07

**Authors:** Maria Joao Azevedo, Maria de Lurdes Pereira, Ricardo Araujo, Carla Ramalho, Egija Zaura, Benedita Sampaio-Maia

**Affiliations:** 1 Faculdade de Medicina Dentária, Universidade do Porto, Portugal.; 2 INEB – Instituto Nacional de Engenharia Biomédica, Universidade do Porto, Portugal.; 3 i3S - Instituto de Investigação e Inovação em Saúde, Universidade do Porto, Portugal.; 4 Academic Center for Dentistry Amsterdam (ACTA), University of Amsterdam and Vrije Universiteit Amsterdam, the Netherlands.; 5 EpiUnit- Instituto de Saúde Pública, Universidade do Porto.; 6 Dept. Medical Biotechnology, College of Medicine and Public Health, Flinders University of South Australia.; 7 Faculdade de Medicina, Universidade do Porto, Portugal.; 8 Centro Hospitalar São João, Porto, Portugal.

**Keywords:** oral fungi, delivery mode, feeding mode, oral colonization, yeasts, Candida, mycobiome

## Abstract

Postnatal acquisition of microorganisms from maternal and environmental sources contributes to the child microbiome development. Several studies showed that the mode of delivery and breastfeeding may have impact on the oral bacterial colonization, however, the influence on oral fungal colonization is still unknown. We performed a systematic literature review on mother to child oral fungi transmission, namely regarding the association between the mode of delivery and breastfeeding in oral yeast colonization. Our analysis revealed no significant differences between the oral mycobiome of breastfed and bottle-fed children. As for the delivery mode, the majority of studies found a relation between fungal colonization and vaginal delivery. *Candida albicans* was the most commonly isolated fungi species. Our analysis suggests that maternal breastfeeding does not seem to influence oral mycology, but vaginal delivery appears to promote oral yeast colonization in early life.

## INTRODUCTION

The human microbiome is a complex ecosystem that varies considerably throughout the body and among individuals [1, 2]. Several factors are known to modulate the development of the infant microbiome, namely host genetics, prenatal environment, delivery mode and postnatal factors, such as antibiotics, environment or diet [3]. It is now known that the maturation of the human microbiome and its homeostasis with the human body might have long-term consequences for health [4].

The microorganisms residing in the oral cavity, and their inevitable inter-relationships, are essential components in changing the balance between health and disease, not only locally but also systemically [5, 6]. From an early stage in life, the human oral cavity comes in contact with a wide variety of microorganisms, and the set of initial colonizers seems to condition the subsequent colonization. These early microbial communities have therefore a major role in the constitution and organization of the adult microbiome and may represent a source of both pathogenic and protective microorganisms in a very early stage of human life.

During delivery there is a significant transmission of microorganisms from the mother to the new-born [3, 7, 8]. This “maternal inoculation” is considered a critical component for the development of infant microbiome [8]. Delivery mode affects significantly the type of transmitted microorganisms; different body sites of children born through vaginal delivery present, five min after birth, bacterial communities similar in composition to vaginal communities, while children born by caesarean-section (C-section) have bacterial communities similar to skin microbiota of mother [7]. The influence of the type of delivery on the establishment of the oral microbiota of the child has yet to be clarified. Holgerson *et al.* [9] showed in healthy three-month-old infants that vaginally delivered infants presented significantly more oral bacterial taxa than infants delivered by C-section, however Chu *et al.* [10] did not find discernible differences in oral community structure or function between six-weeks infants born by C-section or vaginally. Some studies, investigating whether mode of delivery is associated with Mutans streptococci colonization during infancy also found contradictory results [11–14].

Following the delivery mode, the feeding habit may also represent a significant source of oral microorganisms and constitute a microbiome modulator. Breast milk contains bioactive substances that boost microbial colonization and the development of the immune system, as well as growth factors that influence the colonization and maturation of bacteria in the intestinal mucosa [15–17]. Thus, this nourishment imposes itself as one of the most important elements in the postpartum metabolic modulation and immunological programming related to the health of the child [16]. Breast milk includes oral bacteria such as those belonging to *Streptococcus* and *Staphylococcus* [18], with significant increase of *Veillonella*, *Prevotella* and *Leptotrichia* within one to six months after delivery [16]. Accordingly, high amounts of *Streptococcus* spp. were found in both the mother's milk and the infant's saliva [19, 20]. Also, it was shown that the salivary microbiota of three-month-old infants differ among infants exclusively breastfed or fed with artificial milk [21]. Interestingly, a meta-analysis of cross-sectional studies showed that breastfed children are less susceptible to caries than infants fed with artificial milk, suggesting that breastfeeding may protect against dental caries during childhood [22]. Despite this fact, the influence of the feeding mode in the establishment of the gut microbiome is vastly more studied than in the oral microbiome [23, 24].

Although a minority in the oral cavity, fungi play a key role in regulating a healthy balance between microbes and the host by being involved in a wide panoply of chemical, physical and metabolic microenvironment-dependent interactions; for this reason, they are considered a potential “keystone species” [25, 26]. One of the most studied interkingdom interaction is between fungi and bacteria, with symbiotic polymicrobial biofilms reportedly found in oral dysbiosis such as caries, periodontitis, endodontic infections, angular cheilitis and denture stomatitis [5, 27–29]. This symbiotic relation allows, for instance, the presence of bacteria in the mucosa due to the fungal ability of adhering to mucosa, decreases bacterial susceptibility against antibiotic treatment, as shown for *Staphylococcus aureus* and *Candida albicans*, and, additionally, accelerates the recovery of some dominant bacterial species after antimicrobial treatment, as observed in murine models, promoting bacterial resilience [5, 27, 30]. Additionally, it is known that *C. albicans* has the ability of changing its microenvironment, either by influencing its physical properties, such as pH, or by producing secondary metabolites, allowing a selective growing of certain bacterial species and the suppression of some virulence factors, such as the ones produced by *Streptococcus mutans* [25]. Compared to the bacterial component of the microbiota, the mycobiome is poorly studied, perhaps due to the following facts: i) fungi are present in a significantly lower proportion than bacteria, ii) it is difficult to isolate their genetic material and iii) many species are still uncultivable using current methods [26]. In the oral cavity of a healthy individual more than 75 genera of fungi have been found, with the most prevalent being *Candida, Cladosporium, Aureobasidium, Aspergillus* and *Malassezia* spp. [26, 31–34].

Due to the importance of fungi in the oral ecosystem and the lack of knowledge about the acquisition and maturation of the oral mycobiome, the purpose of this study was to perform a systematic literature review on the influence of the type of nourishment and the delivery mode on fungal transmission between mother and infant.

## SYSTEMATIC REVIEW

The search was performed using PubMed database, from April 25 to June 18, 2019. The following combinations of key words were used:

a) (delivery OR cesarean section OR vaginal delivery) AND (yeast OR fungal transmission OR Candida OR fungi OR mycobiota OR mycobiome) AND (infants OR children OR newborn OR neonate OR baby)

b) (feeding mode OR breastfeeding OR bottle feeding) AND (yeast OR fungal transmission OR Candida OR fungi OR mycobiota OR mycobiome) AND (infants OR children OR newborn OR neonate OR baby).

The inclusion criteria used in this systematic review were comparative studies in healthy humans and written in English, related to oral fungi transmission and acquisition. The studies selected must investigate the type of nourishment and delivery mode in relation to the acquisition of the fungi. Both longitudinal studies and cross-sectional studies were included and no restrictions were applied regarding the date of publication of the studies. Systematic reviews were excluded, as also studies regarding exclusively bacteria and virus transmission. Additionally, studies that did not test associations between fungi transmission and the type of delivery or nourishment were excluded. Research papers concerning the use of probiotics and antibiotics in the peri-natal period were not considered, neither studies that did not explore the oral mycobiome.

**Figure 1:**
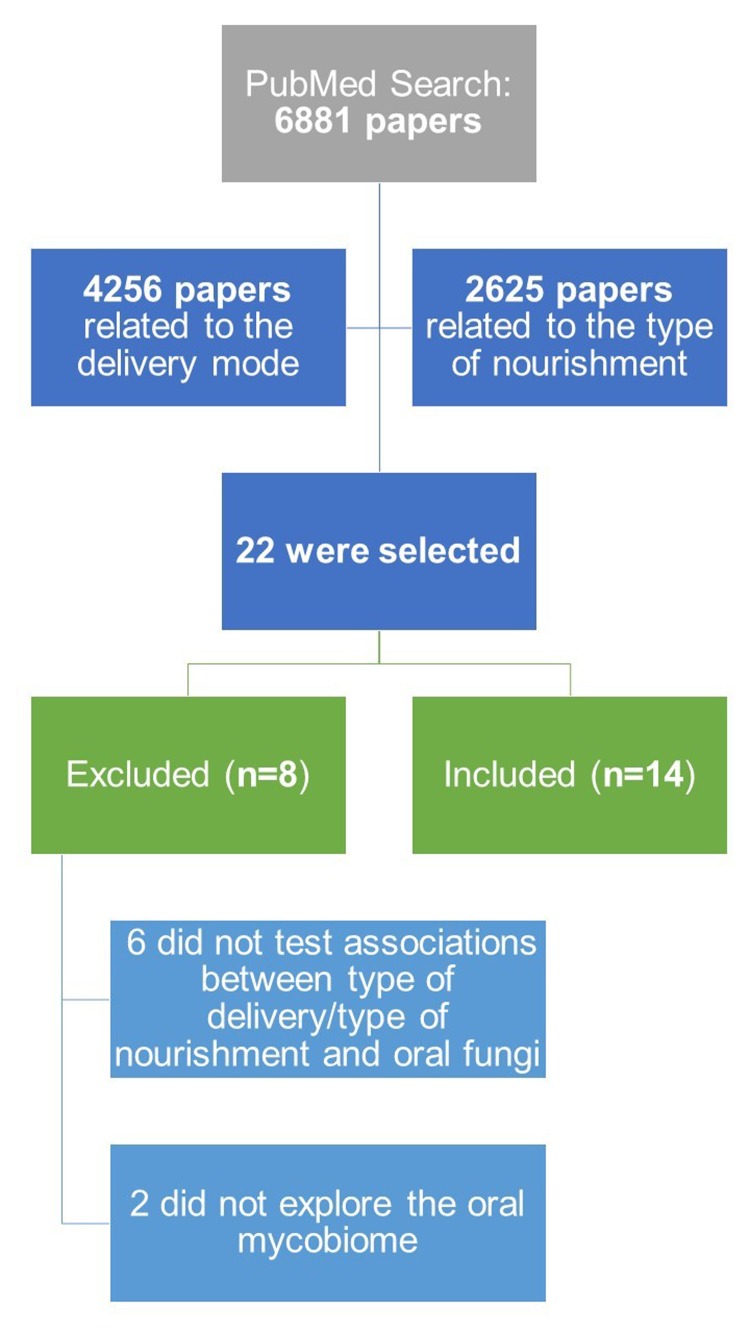
FIGURE 1: Workflow overview of the systematic review.

After the search in PubMed, a total of 6881 papers were retrieved from the query (see **Figure 1**). From this list of scientific papers, titles and abstracts were screened of which 22 papers [35–56] made the final list, after removing duplicated articles. Only 14 articles of these were finally considered suitable for this review [35, 37–39, 41, 42, 44, 46, 50, 51, 53–56], since six articles did not test associations between type of delivery/type of nourishment with the oral mycobiome [36, 40, 43, 45, 48, 52] and two articles did not explore the oral mycobiome [47, 49].

Concerning the type of study, seven of the selected articles were cross-sectional studies [35, 41, 44, 46, 50, 54, 56] and seven were longitudinal studies [37–39, 42, 51, 53, 55].

Regarding the objective of the selected papers, two focused on the colonization in very low weight newborns [37, 38]; one study investigated newborns fungal colonization in intensive care units [55]; one article investigated the interaction between fungi and *Helicobacter pylori* in early colonization [50]; two articles specifically investigated vertical transmission of fungi from mother to child [39, 42]; one article investigated the possibility of an association between the presence of yeasts and the use of pacifiers in children among other factors such as the type of delivery [56]; five articles focused only on the fungal colonization of the mouth of infants in relation with the feeding mode [35, 41, 44, 46, 54]; an article tried to find associations between environmental factors and oral fungi colonization on infants [51]; and one paper investigated the evolution of the mycobiome across body sites during the first month of life [53].

**TABLE 1.** Studies regarding the relation between the feeding mode and the oral mycobiome.

**Samples**	**No. participants**	**% Breast-fed^a^**	**% Bottle-fed^b^**	**Methods**	**Major Findings**	**Ref.**
Swabs from tongue and right buccal mucosa of the infants.	200 infants	N.A.	N.A.	Culture in SAB; Identification by systems API ZYM and API 20C AUX and Boric Acid resistance test	-) No specific pattern between the predominant Candida biotypes and breast feeding.	[46]
Swabs from the dorsum of the tongue, buccal mucosae and palate of the infants.	206 infants	52.9%	4.8%	Culture on SAB agar; Identification by germ-tube test and yeast identification system Microring YT; Scored as: <10 colonies = mild; 11-50 colonies = moderate growth; >50 colonies = heavy growth	-) No significant differences in the frequency of *Candida* isolation or density of growth between infants who were breast-fed, bottle-fed or on both patterns of feeding (p= 0.14); -) Significantly higher frequency of *Candida spp.* isolation from infants who suck pacifiers compared to those who did not (p<0.05).	[41]
Swabs from oral mucosa of cheek, edentulous ridge, dorsum of the tongue and hard palate of the infants	36 infants	0-2 months: 67.31% > 2 months: 15.25%	47.08%	Culture on SAB agar with chloramphenicol (10%); Replication of some cultures on SAB agar; Pure cultures analyzed for specific identification.	-) Yeasts were detected in 58.3% of the children; -) Children who were never breast-fed/breast-fed until two months of age had 15% higher frequency of oral yeasts compared to those who were breast-fed for a longer time; -) No significant association was observed between the prevalence of yeast infection and bottle-feeding; -) The use of a pacifier influenced the colonization and proliferation of yeasts in the oral cavity.	[56]
Tongue swabs from the infants and their mothers; swabs from the skin of the women's nipples and areolae.	169 women and 85 infants	4.71%	35.29%	Culture on SAB agar with chloramphenicol; Identification by considering germ tube production in sterile rabbit serum, the formation of chlamydospores, hyphae and yeasts in corn meal Tween 80 agar, carbohydrate assimilation and fermentation	-) Significant differences in the prevalence of *Candida spp.* related with the feeding mode: 34.55% in breast-fed infants' mouths and in 66.67% of those who were bottle-fed, suggesting a protector role of breastmilk (p<0.05).	[54]
Swabs from dorsal surface of the tongue and mid-palate of the infants	300 infants	N.A.	N.A.	Culture in SAB medium; Identification by germ-tube test, chlamydospore formation on corn meal agar and API 20C AUX system.	-) Differences (p<0.01) in the prevalence of *Candida spp.* carriage between children who were breast-fed and bottle-fed or other fluids and children who were only breast-fed.	[44]
Saliva from the infants.	14 infants	N.A.	N.A.	ChromAgar for analyzing colonies of *Candida spp.*	-No difference in the total *Candida spp*. count between children who are breast/bottle fed and those who are not (p=0.184).	[35]

NA – Information not available in the paper.

SAB – Sabouraud dextrose medium

a Percentage of breast-fed children carriers of oral fungi.

b Percentage of bottle-fed children carriers of oral fungi.

The majority of the articles selected used swabs from the oral sites or saliva. With respect to the time points of collection in the longitudinal studies regarding to the infants, these ranged from within 24 hours after delivery to one year. **Tables 1** and **2** sum up information with respect to the reference number, samples and number of participants, percentage of children carriers of oral fungi who were breastfed/bottle-fed or born by C-section/ vaginal delivery in each study, methodology used to identify fungi and the highlights of the results of each study.

**TABLE 2.** Studies regarding the relation between the delivery mode and the oral mycobiome.

**Samples**	**No. participants**	**% C-section^a^**	**% Vaginally^b^**	**Methods**	**Major Findings**	**Ref.**
Swabs from oropharynx, groin, rectum, perineum and endotracheal tube aspirate andurine of the infants; Breastmilk from the mothers.	146 infants	23.1%	76.9%	Culture on SAB agar; Identification by germ tube production and by sucrose assimilation tests; Germ negative tubes were identified by Uni-Yeast Tek Wheel	-) Significantly more infants with colonization were delivered vaginally compared with the infants without colonization (p<0.05).	[37]
Swabs from cheek, lip and mouth pavement of the neonates; Vaginal secretion of the mothers.	100 mother-infant pairs	28%	72%	Culture on SAB agar; Phenotypical profiling by susceptibility to killer toxins; proteinase research; phospholipase research; serotyping; antifungal susceptibility; Genotyping profiling by RAPD-PCR Reaction.	-) Frequencies of yeast isolation from oral mucosa were 25% and 3.6%, in cases of vaginal and cesarean births.	[39]
Oral and rectal swabs and tracheal aspirates (collected with sterile traps) from infants.	593 neonates	43.23%	56.76%	Culture on SAB agar with 50 mg/mL chloramphenicol and 50 mg/mL gentamicin; Identification by germ tube formation and ChromAgar Candida test, Mycotube test and API ID 32C.	-) Fungal colonization occurred more frequently in neonates born vaginally than in those born after C-section (p=0.053); -) The mouth was the second most frequent site of colonization after the rectum; -) Vaginal delivery was the only factor to significantly increase the risk for early fungal colonization in neonates.	[55]
Oral, rectal, and inguinal samples from infants; maternal vaginal, rectal, hand and oral swabs.	76 mother-infant pairs	51%	49%	*C. albicans* isolates were identified by germ tube production. The rest of the isolates were further identified by the RapidID yeast plus system. Southern Blot Analysis of the isolates, after purification.	-) Infants born vaginally rather than by C-section were at increased risk for early colonization (p=0.009); -) Among all *C. albicans* colonized infants, 41% acquired the organism from the mother by vertical transmission.	[38]
Swabs from the oral cavity of the newborns and oral and vaginal swabs from the mothers.	347 mother-infant pairs	48.15%	51.85%	Culture on YeastGlucose Chloramphenicol (YGC) agar; Pure cultures of yeasts were identified as *Candida spp,* using ChromAgar.	-) The majority of colonized neonates were born vaginally; -) Large quantity of oral *C. albicans* colonies in newborns may suggest correlation between *Candida* colony counts in the vagina of mother and *Candida* colonization in the neonate; -) The only *Candida* species isolated was *C. albicans*; -) All colonized neonates had the same pulsotype of *C. albicans* as their mothers.	[50]
Swabs from the infants' oral and rectal mucosa; swabs from the mothers' vaginal mucosa.	108 mothers and 89 neonates	18.73%	81.27%	Culture on SAB agar; Identification with API 20 CAUX system; Antifungal susceptibility testing by the E-test method.	-) Statistically significant differences between the frequency of oral yeasts isolated from normally-delivered neonates compared to the cesarean group (p = 0.0063).	[42]
Swabs from cheeks and the tongue of the neonates; venous blood (10 ml) and breastmilk (10 ml) from mother.	100 mother-offspring pairs	11%	89%	Samples were cultivated on a selective media Oricult-N semi-quantitative dipslide and scored 0 = no growth; 1 = 10^3^ CFU ml^−1^; 2 = 10^4^ CFU ml^−1^; 3 = 10^5^ CFU ml^−1^	-) Delivery mode was not associated with colonization of the child at four weeks of age (p > 0.05). Colonization was fairly stable until six months of age. Exposure to furry pets and siblings impacted oral Candida.	[51]
Swabs from the oral mucosa, forehead, and anal cavity of infants; swabs from vaginal and anal sites of mothers.	17 infants and 16 mothers	58.82%	41.18%	DNA extraction with Mo Bio Powersoil kit; amplification of the IT2 region by PCR (with modifications), followed by their purification; sequencing with Illumina MiSeq system.	-) For oral mycobiomes, birth mode did not significantly impact the alpha diversity trajectory over time (caesarean section p=0.238; vaginal p=0.873) or beta diversity clustering (p=0.261); -) Caesarean section-born infants have a significantly higher relative abundance of *Candida orthopsilosis* than infants born vaginally (p=0.001).	[53]

NA – Information not available in the paper.

SAB – Sabouraud dextrose medium

a Percentage of children born by C-section carriers of oral fungi.

b Percentage of children born vaginally carriers of oral fungi.

## FEEDING MODE

The literature about the relation between the feeding mode and the oral mycobiome is controversial. From the selected five cross-sectional studies that explored this association, three concluded that there was no difference in the oral mycobiome between the children who were breastfed and children who were bottle-fed. Darwazeh *et al.* [41] studied 206 infants aged from two to eleven months old. There were no significant differences in the frequency of *Candida* species isolation or density of *Candida* spp. growth between infants who were breastfed, bottle-fed or who were on both patterns of feeding (p= 0.14), leading them to conclude that *Candida* spp. are commensals of the oral microbiota and independent of the feeding mode. However, a significantly higher frequency of *Candida* spp. isolates was observed from infants who suck pacifiers compared to those who did not (p<0.05). In accordance with this previous study, a research conducted by Matee *et al.* [46], where swabs from tongue and buccal mucosa were collected from 200 infants (age between six months and two years), demonstrated no specific pattern between the predominant biotypes of *C. albicans* and breastfeeding. The authors admit that results may be due to a small number of biotype clusters used in the study (a total of five) and to the small cohort studied. Additionally, Mattos-Graner *et al.* [56] studied the presence of oral yeasts in a group of 36 children, aged from one to eight months. They described the presence of yeast colonization in 58.3% of the infants and a notorious 15% increase in the frequency of yeasts among children who were never breastfed/only breastfed until two months of age, when compared to those who were breastfed for a longer period. Notwithstanding, no significant differences between the duration of breast-feeding and the frequency of yeasts in the infants were reported (p<0.05), with the only significant factor related to yeasts being the use of pacifier. A more recent study by Neves *et al.* [35] studied saliva (instead of oral swabs) from 14 infants from two to four years grouped into children with or without early childhood caries. It concluded there were no differences in the total *Candida* spp. count between children who were either breast- or bottle-fed (p=0.184). Also, the total number of *Candida* spp. was not influenced by the use of pacifier (p = 0.286). Finally, a study by Stecksen-Blicks *et al.* [51] found that the oral *Candida* colonization was between 11 to 15% during the first year of life, remaining fairly stable during the first six months. These authors concluded that breastfeeding duration was not associated with oral *Candida* colonization of the child.

Contrarily to these previously mentioned results, a research by Zöllner and Jorge [54] found a significant difference in the prevalence of *Candida* in relation with the feeding mode. In this investigation, they sampled tongue swabs from the infants and from their mothers and also swabs from the skin of the women's nipples and areolae (N=169 women and 85 infants, aged from one to five months). Their results showed oral fungal colonization in 34.6% of the breastfed infants (who did not use pacifiers or any other kind of rubber nipples) *versus* 66.7% of the exclusively bottle-fed ones (p<0.05). Moreover, in 81.81% of cases, the *Candida* species coincided in the mouth of the infant and on the breast of the mother, suggesting a transmission between mother breast and child oral cavity or vice versa. Also, there was a higher prevalence of *Candida* spp. on the breast nipple and areolae samples of lactating women than in non-lactating women (p<0.05), explained by moistened, macerated and exposed to constant trauma breast surface that potentiate fungi colonization. The lower oral fungi colonization on breastfed than on non-breastfed children was explained by the authors due to resistance factors present in the breastmilk that may play a protective role in the oral ecosystem of the infant against microorganisms from *Candida* genus. Furthermore, Kadir *et al.* [44] in a cross-sectional study including 64 infants (ages from zero to two years), found significant differences (p<0.01) in the prevalence of candidal carriage between diet groups of Turkish children: 18.5% of children who received breast milk and bottle milk or other fluids were colonized and none of the children who were exclusively breastfed were colonized. The presence of *Candida* was found particularly in children who were fed both breast and bottle milk or fluids sweetened with carbohydrates.

Of notice is that all these studies used classic cultural method for fungi detection, supposedly having similar sensitivities.

## DELIVERY MODE

The relationship between the delivery mode and oral fungi colonization was less controversial and several studies demonstrated this influence.

Firstly, a study by Baley *et al.* [37] (N=146 very low birth weight infants) identified fungi in 26.7% of the very low birth weight infants. The researchers concluded that significantly more infants with fungal colonization were delivered vaginally compared with the infants without colonization (p<0.05). Farmaki *et al.* [55] collected during a twelve-month period and on a weekly basis, oral and rectal swabs, as well as tracheal aspirates, from 593 neonates admitted in a neonatal intensive care unit. Oral colonization was reported in 46 out of 72 colonized neonates. They concluded that fungal colonization occurred 10% more in neonates born vaginally than in those born after C-section (p=0.053) and that vaginal delivery was the only significant risk factor for early colonization. A late colonization may occur due to longer stays in the neonate intensive care unit. Interestingly, very-low birth weight neonates were more frequently colonized by non-*albicans Candida* spp. than neonates with birthweight above 1,500 g, who were mostly colonized by *C. albicans.* In accordance with this study, an investigation by Caramalac *et al.* [39] revealed that the frequencies of yeast isolation from oral mucosa were 25% and 3.6%, in cases of vaginal and cesarean deliveries, respectively. This study was conducted by sampling swabs from mother's oral cavity and vagina, and cheek, lip and the floor of the mouth swabs from the neonates (N=100 pairs), after which they were submitted to phenotyping and genotypic profiling by RAPD-PCR reaction. The authors suggest that vaginal delivery may lead to fungal colonization in neonates, not only due to the direct contact with the vaginal mucosa but also due to the trauma suffered by the newborn while passing through the birth canal, because the results revealed that in only two cases mother and son shared the same genotypic profile of the isolated yeasts. Moreover, Bliss *et al.* [38] gathered a group of 76 mother and very low birth weight newborn-pairs and collected oral, rectal, inguinal, hand and oral samples from newborns and vaginal and rectal cultures from their mothers. *C. albicans* isolates were identified by germ tube production and the remaining isolates were identified by the RapidID yeast plus system. Infants born vaginally (35% of which were colonized) rather than born by C-section (only 10% were colonized) were at increased risk for early colonization (p=0.009) and 41% of the infants shared the same genotype of *C. albicans* with their mothers. Filippidi *et al.* [42] studied a group of 347 mother-infant pairs by collecting vaginal (mother) and oral and rectal swabs (infants). They concluded that the vast majority of colonized neonates were born vaginally and that large quantities of *C. albicans* colonies in the neonate may be correlated with *Candida* colony counts in the mother's vagina. The pulsotype of *C. albicans* was identical between all pairs of mother-infant, contrarily to what was found between mother and infant in the study by Caramalac *et al.* [39]. Siavoshi *et al.* [50] gathered oral and vaginal swabs from 108 mothers and 89 neonates, finding a significantly higher frequency of oral yeasts isolated from vaginally-delivered neonates than from C-section delivered neonates (p <0.01).

Conversely, two recent studies presented dissimilar results. Firstly, a study by Stecksen-Blicks *et al.* [51] collected swabs from cheeks and the tongue from 100 toddlers. Delivery mode was not associated with oral *Candida* colonization of the child at four weeks of age (p> 0.05). Exposure to furry pets and siblings, however, impacted oral *Candida* colonization: the presence of a sibling positively impacted candidal colonization but only at twelve months of age (p<0.05), with almost all colonized infants having a sibling, while having a pet, at three months of age, was related to a lower *Candida* colonization (p<0.05). Furthermore, Ward *et al.* [53] studied 17 infants and 16 mothers and clustered swabs from the oral mucosa, forehead, and anal cavity of infants and from vaginal and anal sites of mothers implying next-generation sequencing. For the oral mycobiome, neither the alpha diversity trajectory over time (p>0.05) nor beta diversity clustering (P=0.261) were influenced by the delivery mode. Nevertheless, it is noteworthy that C-section born infants had a significantly higher relative abundance of oral *Candida orthopsilosis* than infants born vaginally (p=0.001). Curiously, the authors suggest that this difference could be due to antibiotic intake influencing interkingdom relations. Of notice is that this last study used a very different assessment technology from the previous studies and a very low number of mother/child pairs.

## ISOLATED FUNGI

Regarding the identification of oral fungi isolated, *C. albicans* was the most frequently isolated species in numerous studies [35, 37, 41, 42, 44, 53–56]. Other frequently isolated species included *Candida parapsilosis* [37, 41, 44, 53–56], *Candida tropicalis* [37, 53–55], *Candida krusei* [44, 55], *Candida kefyr, Candida famata* [44], *Candida glabrata* [55], *Candida orthopsilosis, Saccharomyces cerevisiae,* and *Cladosporium velox* [53]. Farmaki *et al.* [55] reported the colonization by *Trichosporon sp*. (N=1) and *Candida guilliermondii* (N=1) and three newborns colonized by two yeasts simultaneously (*C. albicans* and *C. glabrata*; *C. albicans* and *Trichosporon sp*.; *C. tropicalis* and *C. lusitaniae*). Caramalac *et al.* [39] reported that *Candida guilliermondii* was the predominant colonizer in newborns, followed by *C. albicans*, albeit this tendency changed during the second week of life, during which predominance of *C. albicans* in vaginally delivered newborns was evident. *Rhodotorula rubra* was only isolated in C-section delivered newborns [39]. It is noteworthy that, in the recent study by Ward *et al.*, the authors highlight that *C. orthopsilosis* was previously characterized as *C. parapsilosis* due to morphological similarities of the two species [53]. This similarity could have misled previous researchers on the subject who based their works on traditional microbiological methods.

As for the *C. albicans* biotypes, Matee *et al.* [46] found more frequently the biotypes J1S (19.5%), A1S (16.0%) and J1R (14.5%). Overall, the prevalence of different fungi isolated and identified may be biased due to the methods employed in each study. The selected papers presented heterogeneous methodology and limited equipment to broadly identify fungal species. Only one paper [53] described a powerful and contemporaneous identification technique (Illumina MiSeq) using an ITS (internal transcribed spacer) region primer (ITS2) and there is a clear lack of research, and consequently of literature, on the subject. More studies using wide-ranged identification tools, such as NGS (next generation sequencing), are required.

## DISCUSSION

**Figure 2:**
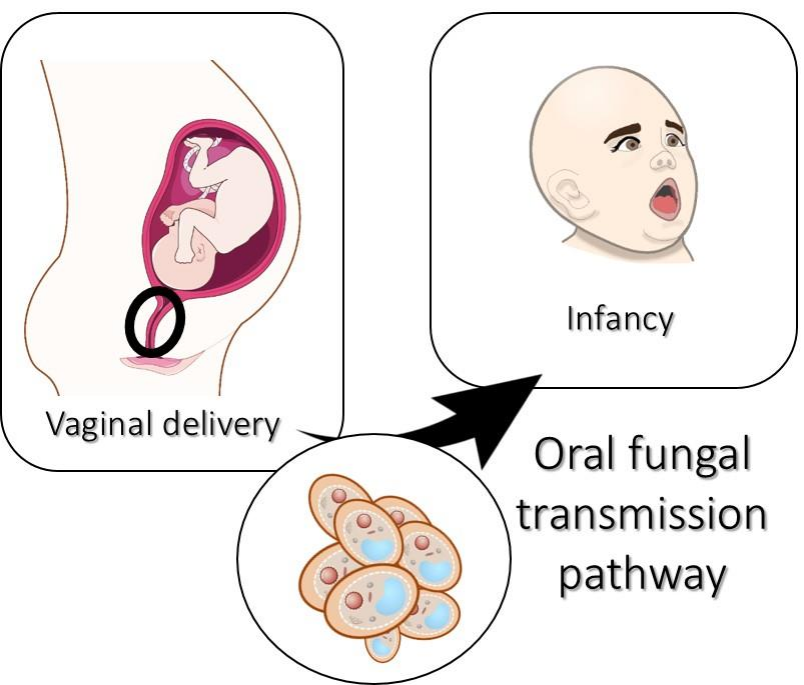
FIGURE 2: Mother-to-child-transmission during vaginal delivery. The figure was partially created using images from Servier Medical Art by Servier, which is licensed under a Creative Commons Attribution 3.0 Unported License; https://smart.servier.com.

Microbiome acquisition in the newborn may represent a crucial process for the microbiome dynamic organization during adulthood. Firstly, our results from the literature review show that delivery mode might be associated with a higher carriage of oral fungi in young age **(Figure 2)** and, on the other hand, in the light of our results, the type of diet of the infant (formula-fed or breast-fed infants) does not seem to have impact on oral yeast carriage, although it is currently recognized that fungi are present in human breastmilk [57]. These results may be explained by the antimicrobial factors of the human milk influencing the oral ecology [58], although further studies are necessary.

Throughout life, the prevalence of fungi varies according to the age group. It is currently recognized that the set of primary oral colonizers strongly influences subsequent colonization, which may lead to more complex and stable ecosystems during adulthood [6, 59]. In this way, the first microbial communities play a fundamental role in the development of the adult microbiota [5, 6]. Immediately after birth, the new born is in contact with a wide variety of microorganisms and can be more easily colonized by maternal microorganisms due to its antigenic tolerance state [60, 61]. Therefore, there is a possibility of vertical microbial transmission of vaginal fungi to the newborn since these are normal colonizers of the vaginal microbiome [62]. Fungi not only contribute with their large cell size and their ability to generate filamentous hyphae to the biofilm structure, but also stimulate the immune system with distinct immunological consequences, helping developing the immune system of the host [26].

The human oral mycobiome includes a great diversity of fungi, however *Candida* is the most prevalent [35, 37, 41, 42, 44, 53, 54]. *C. albicans* was identified in each paper selected for this systematic review, being acknowledged as one of the early colonizers; however, its role in the oral ecosystem is still poorly understood. Moreover, in the literature, the prevalence of fungi in the oral cavity is not consensual. From our review, we verified a lack of studies in this field and a methodological heterogeneity regarding the assessment and identification of the fungal oral microbiota. In addition, most of the methods applied were traditional microbiological methods which, due to their low sensitivity, may preclude the identification of the low prevalence fungi [32]. It is, therefore, important to carry out future studies using more sensitive methods, as reported by Monteiro-da-Silva [33], or molecular biology techniques [32].

## CONCLUSION

Although there is still controversy regarding the influence of the delivery and feeding mode in the acquisition of the oral mycobiome, our results suggest that delivery mode influences the oral yeast carriage in childhood, specifically, vaginal delivery appears to promote oral yeast carriage **(Figure 2)**. On the other hand, maternal breast feeding does not seem to influence oral mycology. More longitudinal studies with comparable sampling, analysis protocols and broad-spectrum identification methods must be performed to have a deeper insight on the underpinning mechanisms of fungal transmission, colonization and the repercussions of the early mycobiome later in life.

## Abbreviations:

C-section: – caesarean-section.

## References

[1] Ding T, Schloss PD (2014). Dynamics and associations of microbial community types across the human body.. Nature.

[2] Huttenhower C, Gevers D, Knight R, Abubucker S, Badger JH, Chinwalla AT, Creasy HH, Earl AM, FitzGerald MG, Fulton RS, Giglio MG, Hallsworth-Pepin K, Lobos EA, Madupu R, Magrini V, Martin JC, Mitreva M, Muzny DM, White O (2012). Structure, function and diversity of the healthy human microbiome.. Nature.

[3] Tamburini S, Shen N, Wu HC, Clemente JC (2016). The microbiome in early life: implications for health outcomes.. Nat Med.

[4] DeWeerdt S (2018). How baby's first microbes could be crucial to future health.. Nature.

[5] Sampaio-Maia B, Caldas IM, Pereira ML, Perez-Mongiovi D, Araujo R (2016). The Oral Microbiome in Health and Its Implication in Oral and Systemic Diseases.. Adv Appl Microbiol.

[6] Sampaio-Maia B, Monteiro-Silva F (2014). Acquisition and maturation of oral microbiome throughout childhood: An update.. Dent Res J.

[7] Dominguez-Bello MG, Costello EK, Contreras M, Magris M, Hidalgo G, Fierer N, Knight R (2010). Delivery mode shapes the acquisition and structure of the initial microbiota across multiple body habitats in newborns.. Proc Natl Acad Sci U S A.

[8] Power ML, Quaglieri C, Schulkin J (2017). Reproductive Microbiomes: A New Thread in the Microbial Network.. Reprod Sci.

[9] Lif Holgerson P, Harnevik L, Hernell O, Tanner AC, Johansson I (2011). Mode of birth delivery affects oral microbiota in infants.. J Dent Res.

[10] Chu DM, Ma J, Prince AL, Antony KM, Seferovic MD, Aagaard KM (2017). Maturation of the infant microbiome community structure and function across multiple body sites and in relation to mode of delivery.. Nat Med.

[11] Li Y, Caufield PW, Dasanayake AP, Wiener HW, Vermund SH (2005). Mode of delivery and other maternal factors influence the acquisition of Streptococcus mutans in infants.. J Dent Res.

[12] Pattanaporn K, Saraithong P, Khongkhunthian S, Aleksejuniene J, Laohapensang P, Chhun N, Chen Z, Li Y (2013). Mode of delivery, mutans streptococci colonization, and early childhood caries in three- to five-year-old Thai children.. Community Dent Oral Epidemiol.

[13] Thakur R, Singh MG, Chaudhary S, Manuja N (2012). Effect of mode of delivery and feeding practices on acquisition of oral Streptococcus mutans in infants.. Int J Paediatr Dent.

[14] Ubeja RG, Bhat C (2016). Mode of Delivery and Its Influence on the Acquisition of Streptococcus mutans in Infants.. Int J Clin Pediatr Dent.

[15] Gomez-Gallego C, Collado MC, Perez G, Ilo T, Jaakkola UM, Bernal MJ, Periago MJ, Frias R, Ros G, Salminen S (2014). Resembling breast milk: influence of polyamine-supplemented formula on neonatal BALB/cOlaHsd mouse microbiota.. Br J Nutr.

[16] Cabrera-Rubio R, Collado MC, Laitinen K, Salminen S, Isolauri E, Mira A (2012). The human milk microbiome changes over lactation and is shaped by maternal weight and mode of delivery.. Am J Clin Nutr.

[17] Al-Shehri SS, Sweeney EL, Cowley DM, Liley HG, Ranasinghe PD, Charles BG, Shaw PN, Vagenas D, Duley JA, Knox CL (2016). Deep sequencing of the 16S ribosomal RNA of the neonatal oral microbiome: a comparison of breast-fed and formula-fed infants.. Sci Rep.

[18] Hunt KM, Foster JA, Forney LJ, Schutte UM, Beck DL, Abdo Z, Fox LK, Williams JE, McGuire MK, McGuire MA (2011). Characterization of the diversity and temporal stability of bacterial communities in human milk.. PLoS One.

[19] Biagi E, Quercia S, Aceti A, Beghetti I, Rampelli S, Turroni S, Faldella G, Candela M, Brigidi P, Corvaglia L (2017). The Bacterial Ecosystem of Mother's Milk and Infant's Mouth and Gut.. Front Microbiol.

[20] Dave V, Street K, Francis S, Bradman A, Riley L, Eskenazi B, Holland N (2016). Bacterial microbiome of breast milk and child saliva from low-income Mexican-American women and children.. Pediatr Res.

[21] Holgerson PL, Vestman NR, Claesson R, Ohman C, Domellof M, Tanner AC, Hernell O, Johansson I (2013). Oral microbial profile discriminates breast-fed from formula-fed infants.. J Pediatr Gastroenterol Nutr.

[22] Avila WM, Pordeus IA, Paiva SM, Martins CC (2015). Breast and Bottle Feeding as Risk Factors for Dental Caries: A Systematic Review and Meta-Analysis.. PloS one.

[23] Pannaraj PS, Li F, Cerini C, Bender JM, Yang S, Rollie A, Adisetiyo H, Zabih S, Lincez PJ, Bittinger K, Bailey A, Bushman FD, Sleasman JW, Aldrovandi GM (2017). Association Between Breast Milk Bacterial Communities and Establishment and Development of the Infant Gut Microbiome.. JAMA Pediatrics.

[24] Lewis ZT, Mills DA (2017). Differential Establishment of Bifidobacteria in the Breastfed Infant Gut.. Nestle Nutr Inst Workshop Ser.

[25] Krom BP, Kidwai S, Ten Cate JM (2014). Candida and other fungal species: forgotten players of healthy oral microbiota.. J Dent Res.

[26] Baker JL, Bor B, Agnello M, Shi W, He X (2017). Ecology of the Oral Microbiome: Beyond Bacteria.. Trends Microbiol.

[27] O'Donnell LE, Millhouse E, Sherry L, Kean R, Malcolm J, Nile CJ, Ramage G (2015). Polymicrobial Candida biofilms: friends and foe in the oral cavity.. FEMS Yeast Res.

[28] Pereira D, Seneviratne C, Koga-Ito C, Samaranayake L (2018). Is the oral fungal pathogen Candida albicans a cariogen?. Oral Dis.

[29] Morse DJ, Wilson MJ, Wei X, Bradshaw DJ, Lewis MAO, Williams DW (2019). Modulation of Candida albicans virulence in in vitro biofilms by oral bacteria.. Lett Appl Microbiol.

[30] Harriott MM, Noverr MC (2009). Candida albicans and Staphylococcus aureus form polymicrobial biofilms: effects on antimicrobial resistance.. Antimicrob Agents Chemother.

[31] Dupuy AK, David MS, Li L, Heider TN, Peterson JD, Montano EA, Dongari-Bagtzoglou A, Diaz PI, Strausbaugh LD (2014). Redefining the human oral mycobiome with improved practices in amplicon-based taxonomy: discovery of Malassezia as a prominent commensal.. PLoS One.

[32] Ghannoum MA, Jurevic RJ, Mukherjee PK, Cui F, Sikaroodi M, Naqvi A, Gillevet PM (2010). Characterization of the oral fungal microbiome (mycobiome) in healthy individuals.. PLoS Pathog.

[33] Monteiro-da-Silva F, Araujo R, Sampaio-Maia B (2014). Interindividual variability and intraindividual stability of oral fungal microbiota over time.. Med Mycol.

[34] Monteiro-da-Silva F, Sampaio-Maia B, Pereira Mde L, Araujo R (2013). Characterization of the oral fungal microbiota in smokers and non-smokers.. Eur J Oral Sci.

[35] Neves A, Lobo L, Pinto K, Pires E, Requejo M, Maia L, Antonio A (2015). Comparison between Clinical Aspects and Salivary Microbial Profile of Children with and without Early Childhood Caries: A Preliminary Study.. J Clin Pediatr Dent.

[36] Alteras I, Aryeli J (1980). The incidence of Candida albicans in the last day of pregnancy and the first days of the new born.. Mycopathologia.

[37] Baley JE, Kliegman RM, Boxerbaum B, Fanaroft AA (1986). Fungal Colonization in the Very Low Birth Weight Infant.. Pediatrics.

[38] Bliss JM, Basavegowda KP, Watson WJ, Sheikh AU, Ryan RM (2008). Vertical and Horizontal Transmission of Candida albicans in Very Low Birth Weight Infants Using DNA Fingerprinting Techniques.. Pediatr Infect Dis J.

[39] Caramalac DA, da Silva Ruiz L, de Batista GC, Birman EG, Duarte M, Hahn R, Paula CR (2007). Candida isolated from vaginal mucosa of mothers and oral mucosa of neonates: occurrence and biotypes concordance.. Pediatr Infect Dis J.

[40] Chow BD, Reardon JL, Perry EO, Laforce-Nesbitt SS, Tucker R, Bliss JM (2016). Host Defense Proteins in Breast Milk and Neonatal Yeast Colonization.. J Hum Lact.

[41] Darwazeh AM, al-Bashir A (1995). Oral candidal flora in healthy infants.. J Oral Pathol Med.

[42] Filippidi A, Galanakis E, Maraki S, Galani I, Drogari-Apiranthitou M, Kalmanti M, Mantadakis E, Samonis G (2014). The effect of maternal flora on Candida colonisation in the neonate.. Mycoses.

[43] Gabriel I, Olejek A, Stencel-Gabriel K, Wielgos M (2018). The influence of maternal vaginal flora on the intestinal colonization in newborns and 3-month-old infants.. J Matern Fetal Neonatal Med.

[44] Kadir T, Uygun B, Akyuz S (2005). Prevalence of Candida species in Turkish children: relationship between dietary intake and carriage.. Arch Oral Biol.

[45] Leibovitz E, Livshiz-Riven I, Borer A, Taraboulos-Klein T, Zamir O, Shany E, Melamed R, Rimon O-F, Bradenstein R, Chodick G, Golan A (2013). A prospective study of the patterns and dynamics of colonization with Candida spp. in very low birth weight neonates.. Scand J Infect Dis.

[46] Matee MI, Samaranayake LP, Scheutz F, Simon E, Lyamuya EF, Mwinula J (1996). Biotypes of oral Candida albicans isolates in a Tanzanian child population.. APMIS.

[47] Pinhat EC, Borba MGS, Ferreira ML, Ferreira MA, Fernandes RK, Nicolaou SK, Okamoto CT, O. Neto CF (2012). Colonização fúngica em recém-natos de muito baixo peso: um estudo de coorte.. J Pediatr.

[48] Ross JM, Needham JR (1980). Genital flora during pregnancy and colonization of the newborn.. J R Soc Med.

[49] Schei K, Avershina E, Oien T, Rudi K, Follestad T, Salamati S, Odegard RA (2017). Early gut mycobiota and mother-offspring transfer.. Microbiome.

[50] Siavoshi F, Taghikhani A, Malekzadeh R, Sarrafnejad A, Kashanian M, Jamal AS, Saniee P, Sadeghi S, Sharifi AH (2013). The Role of Mother's Oral and Vaginal Yeasts in Transmission of Helicobacter Pylori to Neonates.. Arch Iran Med.

[51] Stecksen-Blicks C, Granstrom E, Silfverdal SA, West CE (2015). Prevalence of oral Candida in the first year of life.. Mycoses.

[52] Waggoner-Fountain LA, Walker MW, Hollis RJ, Pfaller MA, Ferguson JE, Wenzel RP, Donowitz LG (1996). Vertical and horizontal transmission of unique Candida species to premature newborns.. Clin Infect Dis.

[53] Ward TL, Dominguez-Bello MG, Heisel T, Al-Ghalith G, Knights D, Gale CA (2018). Development of the Human Mycobiome over the First Month of Life and across Body Sites.. mSystems.

[54] Zöllner MSAdC, Jorge AOC (2003). Candida spp. occurrence in oral cavities of breastfeeding infants and in their mothers' mouths and breasts.. Braz Oral Res.

[55] Farmaki E, Evdoridou J, Pouliou T, Bibashi E, Panagopoulou P, Filioti J, Benos A, Sofianou D, Kremenopoulos G, Roilides E (2007). Fungal colonization in the neonatal intensive care unit: risk factors, drug susceptibility, and association with invasive fungal infections.. Am J Perinatol.

[56] Mattos-Graner RO, de Moraes AB, Rontani RM, Birman EG (2001). Relation of oral yeast infection in Brazilian infants and use of a pacifier.. ASDC J Dent Child.

[57] Boix-Amoros A, Martinez-Costa C, Querol A, Collado MC, Mira A (2017). Multiple Approaches Detect the Presence of Fungi in Human Breastmilk Samples from Healthy Mothers.. Sci Rep.

[58] Timby N, Domellof M, Holgerson PL, West CE, Lonnerdal B, Hernell O, Johansson I (2017). Oral Microbiota in Infants Fed a Formula Supplemented with Bovine Milk Fat Globule Membranes - A Randomized Controlled Trial.. PLoS One.

[59] Gronlund MM, Lehtonen OP, Eerola E, Kero P (1999). Fecal microflora in healthy infants born by different methods of delivery: permanent changes in intestinal flora after cesarean delivery.. J Pediatr Gastroenterol Nutr.

[60] Mold JE, Michaelsson J, Burt TD, Muench MO, Beckerman KP, Busch MP, Lee TH, Nixon DF, McCune JM (2008). Maternal alloantigens promote the development of tolerogenic fetal regulatory T cells in utero.. Science.

[61] Zaura E, Nicu EA, Krom BP, Keijser BJ (2014). Acquiring and maintaining a normal oral microbiome: current perspective.. Front Cell Infect Microbiol.

[62] Bradford LL, Ravel J (2017). The vaginal mycobiome: A contemporary perspective on fungi in women's health and diseases.. Virulence.

